# Investigating the association between obesity and asthma in 6- to 8-year-old Saudi children: a matched case–control study

**DOI:** 10.1038/npjpcrm.2014.4

**Published:** 2014-06-05

**Authors:** Mahmoud Nahhas, Raj Bhopal, Chantelle Anandan, Rob Elton, Aziz Sheikh

**Affiliations:** 1 Allergy and Respiratory Research Group, Centre for Population Health Sciences, Medical School, The University of Edinburgh, Edinburgh, UK; 2 Bruce and John Usher Professor of Public Health, Centre for Population Health Sciences, Medical School, The University of Edinburgh, Edinburgh, UK; 3 Centre for Population Health Sciences, Medical School, The University of Edinburgh, Edinburgh, UK; 4 Division of General Internal Medicine and Primary Care, Brigham and Women’s Hospital/Harvard Medical School, Boston, MA, USA

## Abstract

**Background:**

Previous studies have demonstrated an association between obesity and asthma, but there remains considerable uncertainty about whether this reflects an underlying causal relationship.

**Aims:**

To investigate the association between obesity and asthma in pre-pubertal children and to investigate the roles of airway obstruction and atopy as possible causal mechanisms.

**Methods:**

We conducted an age- and sex-matched case–control study of 1,264 6- to 8-year-old schoolchildren with and without asthma recruited from 37 randomly selected schools in Madinah, Saudi Arabia. The body mass index (BMI), waist circumference and skin fold thickness of the 632 children with asthma were compared with those of the 632 control children without asthma. Associations between obesity and asthma, adjusted for other potential risk factors, were assessed separately in boys and girls using conditional logistic regression analysis. The possible mediating roles of atopy and airway obstruction were studied by investigating the impact of incorporating data on sensitisation to common aeroallergens and measurements of lung function.

**Results:**

BMI was associated with asthma in boys (odds ratio (OR)=1.14, 95% confidence interval (CI), 1.08–1.20; adjusted OR=1.11, 95% CI, 1.03–1.19) and girls (OR=1.37, 95% CI, 1.26–1.50; adjusted OR=1.38, 95% CI, 1.23–1.56). Adjusting for forced expiratory volume in 1 s had a negligible impact on these associations, but these were attenuated following adjustment for allergic sensitisation, particularly in girls (girls: OR=1.25; 95% CI, 0.96–1.60; boys: OR=1.09, 95% CI, 0.99–1.19).

**Conclusions:**

BMI is associated with asthma in pre-pubertal Saudi boys and girls; this effect does not appear to be mediated through respiratory obstruction, but in girls this may at least partially be mediated through increased risk of allergic sensitisation.

## Introduction

The substantial parallel increases in the prevalence of obesity^1–5^ and asthma^6–8^ observed over recent decades have led to the suggestion that obesity may be causally implicated in the risk of developing asthma.^9–11^ This line of enquiry began with the study of female nurses enrolled in the Nurses’ Health Study cohort, which found a strong, dose-dependent relationship between an increase in body mass index (BMI) and adult-onset asthma.^12^ Investigations into the association between obesity and asthma have subsequently been extended to both male and female adults,^13^ adolescents^14–16^ and young children,^17^ other populations of European origin^9,18–20^ and some other ethnic groups.^17^ Overall, this body of work has confirmed the original observation—namely that obesity is associated with an increased risk of developing asthma,^15^ particularly in post-pubertal females.^21^ What is, however, far less clear is whether this reflects a causal relationship between the two and, if so, what the underlying mechanisms might be.

Judgements on causal inference can be greatly aided by studying biological mechanisms^22^ and it is therefore welcome that the epidemiological evidence has triggered a substantial body of mechanistic work in both animal and human populations.^23,24^ Broadly speaking, this work has converged on studying the role of common genes,^25,26^ fetal programming,^27^ sex hormones,^11,27,28^ respiratory obstruction,^29^ bronchial hyper-reactivity^21^ and changes in immunological and inflammatory responses.^11,26,27^ This body of work has, however, thus far failed to generate any clear insights into possible biological pathways, possibly reflecting the fact that different mechanisms may be implicated in different population subgroups. It has therefore been suggested that age- and gender-specific investigations be undertaken in an attempt to progress understanding on possible causal pathways.^21^

We sought to contribute to this important area of enquiry by undertaking the first study of obesity and asthma in a Middle Eastern population. We investigated the association between obesity and asthma in pre-pubertal school-aged children, undertaking separate analyses in boys and girls, with a view to understanding the possible aetiological roles of physiological (i.e., respiratory obstruction) and immunological (i.e., allergic sensitisation) mechanisms.

## Materials and Methods

### Ethical considerations

Permission was obtained from the education authorities in Madinah (General Directors) and School Health Departments in the Ministry of Education, Riyadh, Saudi Arabia, and from head-teachers of participating schools. Parents gave their signed, informed consent to their child’s participation. All data had identifiers removed to minimise the risk of inadvertently breaching confidentiality.

### Design

We conducted an age- and sex-matched case–control study of 1,264 schoolchildren with and without asthma. Children aged 6–8 years were recruited from 37 randomly selected schools in Madinah, Saudi Arabia. These children were identified from our linked cross-sectional study of over 5,000 children in whom we found an asthma prevalence of 23.6% (95% confidence interval (CI), 21.3, 26.0); this group of children provided a sampling frame for this investigation.^30^ For this phase of work, we compared BMI, triceps skin fold thickness (TSFT) and waist circumferences (WCs) in children with a history of symptoms indicative of asthma (cases) with individually age- and sex-matched children without such a history (controls), factoring respiratory function and allergic sensitisation into the analyses. Pilot work confirmed the feasibility of undertaking this research.

### Setting

Madinah is a city in the west of Saudi Arabia, with an area of 589 km^2^, which lies approximately 190 km from the Red Sea coast. It has a population of over 1.5 million people, approximately 5% of the total of 21.4 million residents in Saudi Arabia.^31^

### Recruitment of schools and students

Lists of boys’ and girls’ primary schools were obtained from the General Directorate of Education of the Madinah region. Schools were then stratified according to the five geographical areas of Madinah and students’ gender and a random sample of 37 schools (i.e., 8 girls’ and 29 boys’ schools) were approached. We invited the schools to participate with a letter of explanation to the head-teachers outlining the purpose of the study and the procedures that were to be employed.

Eligible children were those aged 6–8 years who had been resident in Madinah for at least 1 year prior to the start of the study. All eligible subjects were invited to participate via a letter and a consent form sent by the school to their parents.

### Identification of cases and controls

The parents of all recruited children were asked to complete a questionnaire incorporating validated questions from the International Study of Asthma and Allergies in Childhood (ISAAC)^32^ that had previously been adapted for use in Arab populations.^33^ Cases were defined as children who were identified by parents as those who had ‘ever had wheeze’;^30,34–36^ controls were defined as those children, matched for age and sex, who had according to their parents ‘never had wheeze’ as reported using the same instrument.

### Measures of obesity

Our primary measure of obesity was BMI,^37^ but, in addition, we measured weight, TSFT and WC. Children were asked to wear normal light clothing during measurements and to take off their shoes. Measurements of height to enable calculation of BMI (which was calculated as weight (kg) divided by the square of height in metres (kg/m^2^)), weight, TSFT and WC were recorded in the schools by trained members of the health-care team who were unaware of whether children had asthma or not. Height was measured with a portable stadiometer (SECA Leicester height measure, SECA, Birmingham, UK) using the method described by Tanner *et al.*^38^ Measurements were made to the nearest 0.1 cm.

Children were weighed and measured separately from their classmates. Weight was measured using SECA Mechanical Column Scales, with measurements being recorded to the nearest 0.1 kg.

We used BMI percentiles for girls and boys per age as a measure of standardised weight. As there are no reference data available for Saudi children, we used the Center for Disease Control and Prevention (CDC) BMI-for-age growth charts for girls and boys.^39^ According to these charts, underweight was defined as a BMI less than the 5th centile, healthy weight was defined as a BMI between the 5th and the 85th centile, overweight was defined as a BMI between the 85th and 94th centiles and obese was defined as a BMI equal to or greater than the 95th centile.

TSFT was measured three times to the nearest millimetre with a Holtain Skinfold Caliper (Holtain, Crymych, UK) on the right arm. The triceps skin fold locus was defined as being halfway between the acromion and olecranon on the back of the arm measured with the elbow bent.^40,41^ TSFT was used mainly to determine relative obesity and the percentage of body fat.^40,41^

WC was measured to the nearest centimetre with a Lufkin flexible steel tape measure (Apex Tool Group, Sparks, MD, USA), with children in the standing position after gentle expiration. The following anatomical landmarks were used: laterally, midway between the lowest portion of the rib cage and iliac crest; and anteriorly, midway between the xiphoid process of the sternum and the umbilicus.^42^

### Potential confounders

The parental questionnaire provided information on the following potential confounders:

- Parental age
- Parental education
- Parental smoking status
- Number of siblings
- Place of birth
- Breastfeeding
- Medications administered (i.e., antibiotics and paracetamol)
- Exposure to pets and farm animals
- Food and other lifestyle factors

### Measurement of explanatory variables

We measured lung function and undertook skin prick tests to common aeroallergens to investigate whether the presence of respiratory obstruction or allergic sensitisation could help explain any possible associations uncovered.

#### Lung function tests

Lung function testing was performed in schools by trained members of the research team. All spirometric measurements were taken in a sitting position using a Vitalograph Pneumotrac spirometer (Vitalograph, Buckingham, UK). England The best of at least three technically acceptable values for forced expiratory volume in 1 s (FEV_1_), forced vital capacity (FVC), (FEV_1_%) FEV_1_/FVC ratio and peak expiratory flow were selected.^43–45^

#### Skin prick tests

Skin prick tests were undertaken using the volar aspect of the forearm, the skin having first been marked with a pen. Thereafter, one drop of each solution to be tested was put on the skin beside the respective mark. A panel of eight allergens (i.e., cat, *Dermatophagoides farinae*, *Dermatophagoides pteronyssinus*, grass mix, Rye, tree mix (early blossoming), tree mix (mid-blossoming) and weed mix) were used. Histamine 10 mg/ml and 50% glycerine were used as positive and negative controls, respectively. The size of the weal was recorded as the mean of the greatest diameter and the diameter perpendicular to its midpoint. Recordings were taken after 15 min and a mean diameter of 3 mm or greater than the negative control was regarded as positive.^46^

### Statistical methods

All statistical analyses were carried out using Statistical Package for Social Sciences (SPSS, Chicago, IL, USA), version 16.0, for Windows and Confidence Interval Analysis (CIA) software (University of Southampton, Southampton, UK).^47^ All analyses were undertaken separately for boys and girls because the literature suggested that we might find differing degrees of association between obesity and asthma.^21,28^ Univariate comparisons between the matched cases and controls were carried out by paired *t*-test variables and Wilson’s method for binary variables. The differences in measures of obesity between the matched pairs of children were tested for variation between schools, separately for each sex, by one-way analysis of variance. There were no significant differences between schools in any of the four measures, suggesting no important cluster effects, and all further analyses were therefore carried out without any adjustment for school effects. Multiple conditional logistic regression was used to investigate the relationship between asthma and explanatory variables, adjusted for confounders. In the regression models, only those variables that differed significantly between cases and controls were included as covariates.

### Sample size calculations

Our preliminary work^30^ suggested that the prevalence of asthma was around 15% and therefore a sample of around 5,000 children needed to be recruited into the cross-sectional study in order to recruit sufficient numbers of cases (⩾600) to give 80% power to detect a mean difference of around 0.4 in BMI between cases and the same number of controls based on a s.d. of three units for BMI.

## Results

### Recruitment of schools

All 37 schools that were approached agreed to participate.

### Characteristics of cases and controls

Data were collected on 388 matched pairs of boys and 244 matched pairs of girls. Table 1 summarises the main characteristics of cases and controls among boys and girls. The demographic and lifestyle variables that differed between cases and controls in males were birth weight, smoking habits of the father, breastfeeding, taking of paracetamol and antibiotics during the first year of life, taking of paracetamol in the previous 12 months and the frequency of trucks passing near the home. In girls, demographic and lifestyle variables that differed between cases and controls were smoking habits of the father, breastfeeding, exposure to farm animals during the first year of life, taking paracetamol during the previous 12 months, exposure to a cat during the previous 12 months, the type of air conditioning system at home and meat and nut consumption.

### Univariate analysis

Table 2 shows the unadjusted effect of each of the obesity measures on the risk of having asthma in boys and girls. All four obesity measures (BMI, weight, TSFT and WC) were found to be positively associated with asthma in both boys and girls. The odds ratios in girls were consistently higher than those in boys.

### Multivariate analysis

Table 3 shows the effect of BMI on the risk of asthma in boys and girls, adjusted for relevant potential confounding sociodemographic and lifestyle variables. Only those variables showing univariate associations with asthma were included in each model. These analyses showed that BMI was still strongly associated with asthma after adjustment for these covariates in both boys and girls. The adjusted odds ratio was higher in girls than in boys.

### Investigation of possible causal pathways

There was—in both sexes—strong evidence that children with asthma had greater respiratory obstruction and were more sensitised to aeroallergens in comparison with those with no history of asthma. Table 4 shows the impact of factoring measures of respiratory obstruction and this reveals a negligible impact on the associations between BMI and asthma in boys and girls. Table 4 also reveals the impact of factoring the presence of allergic sensitisation into the analysis and this in contrast shows a more marked reduction in the associations in girls, although a more modest impact in boys.

## Discussion

### Main findings

This large case–control study has demonstrated consistent associations between measures of overall, central and peripheral obesity, and asthma in boys and girls. In keeping with previous work,^17,20^ we found a stronger association in girls than in boys, but unlike other studies we observed this difference in the context of studying pre-pubertal children. Focusing on BMI, we then demonstrated that this association was still present after adjusting for a wide range of potential demographic and lifestyle-related confounding factors. In an attempt to investigate whether respiratory obstruction and/or allergic sensitisation may lie on the causal pathway between obesity and asthma,^11,26^ we adjusted separately and then combined for their effects and found that respiratory obstruction had only a very modest impact, but that the association was more attenuated when adjusting for sensitisation, particularly in girls. Adjusting simultaneously for both lung function and sensitisation did not further attenuate the relationship, suggesting that respiratory obstruction is unlikely to be an important mechanism but that obesity may at least partially exert its effects by increasing the risk of allergic sensitisation. Overall, this study therefore adds to the increasing body of evidence implicating obesity as a risk factor for the development of asthma showing that this relationship also holds true in a population of Middle Eastern origin and provides important pointers into how this relationship may or may not be mediated in this population.

### Strengths and limitations of this study

Key strengths of this work include the fact that we conducted a large, adequately powered, investigation, our use of a validated and extensively used questionnaire to identify cases and controls, careful measurements of a range of measures of obesity^17^ by trained personnel who were blinded to case status the measurement of, and adjustments for, a range of potential confounders and the *a priori* decision to undertake separate analyses in boys and girls.^21,28^ In addition, we sought to extend previous work by investigating two important possible causal mechanisms.

The main limitations of this work are those inherent in case–control studies—namely the risk of residual confounding, recall bias and an inability to establish a temporal relationship between the onset of obesity and the subsequent development of asthma; reverse causality therefore remains a possibility. Moreover, the identification of cases was based on responses to the ISAAC questionnaire and this may have been affected by parental behaviours and diagnostic bias; although a validated culturally appropriate questionnaire was used, there remains therefore the possibility of misclassification errors. If present, this is likely to have been operating in a non-differential manner, which would have resulted in blunting the associations between obesity and asthma rather than creating spurious associations. Future work should seek to build on this by using more objective assessments to diagnose asthma, although given the numbers of subjects who need to be studied this may well prove financially and logistically challenging.

### Interpretation of findings in relation to previously published research

Our study is consistent with an increasing number of epidemiological studies that have reported a positive association between obesity and the risk of developing childhood asthma.^17,20^ As with other investigators,^17^ we have found that this association was stronger in girls. Whether this sex difference is simply due to the higher levels of obesity observed in female cases (Table 1) or due to difference in body fat distribution—particularly centrally, where it is believed to exert the greatest inflammatory effect,^48,49^ as reflected by the larger WC and prevalence of sensitisation in females with asthma (Table 1)—and/or associated biochemical responses is unclear.^48,49^ The fact that sensitisation was a strong risk factor for asthma confirms the findings of a number of previous epidemiological investigations.^46^

There is a limited body of evidence investigating the relationship between obesity and allergic sensitisation, and the available evidence has been noted to be inconsistent and inconclusive.^21^ A large and important recent Swedish birth cohort study found that increased BMI at age 7 was associated with an increased risk of allergic sensitisation to aeroallergens and the development of childhood asthma, but that asthma risk was not increased if the increased BMI was confined to the first 4 years of life.^50^ It is thus likely that any possible relationship between obesity and allergic sensitisation is strongly influenced by age. Sex is also important as recent work has also demonstrated that levels of the adipocyte-derived hormone leptin are closely correlated with BMI in early life, and that leptin levels tend to be higher in girls than in boys,^51^ which may explain why a stronger and more consistent relationship is observed between BMI and asthma in girls.^21^ Future work should now aim to study carefully characterised populations and also progress mechanistic lines of enquiry through measurement of key biomarkers including the adipokines adiponectin and leptin, other obesity-related hormones such as insulin and neuropepeides, and the pro-inflammatory cytokines tumour necrosis factor, interleukin-6 and interleukin-1B;^27^ it is also important to investigate whether obesity reduces immunological tolerance through downregulation of interleukin-10 secretion and study the combined impact of these changes on regulatory T lymphocytes (Tregs).^52^

### Implications for future research, policy and practice

The findings of the present study may be valuable for public health authorities as it provides additional evidence of the considerable adverse risks associated with obesity. Although causality cannot be proven by this or indeed any other epidemiological investigation, the findings from this work, when combined with an increasing number of studies worldwide,^12,13,17,20^ provide increasing evidence of a causal relationship between obesity and asthma. This then adds weight to the argument to consider and investigate the role of interventions to prevent/tackle obesity early on in life and thereby possibly reduce the risk of developing asthma. Even if such causality has not been unequivocally proven, public health authorities should consider applying the ‘precautionary principle’^53^ and undertake initiatives for reducing obesity among children. Reduction of obesity in children will offer benefits in the general health status, as childhood obesity is a well-established risk factor for several other diseases.^54^ Moreover, given the high prevalence of obesity and asthma, school health departments should consider focussing attention on establishing systems for monitoring obesity and asthma among schoolchildren.

## Conclusions

In conclusion, our large matched case–control study provides the first evidence demonstrating an association between obesity and asthma in pre-pubertal male and female Saudi children. Our investigation of key causal pathways suggests that this relationship may indeed be causal, being at least partially mediated through the pro-inflammatory effects of central obesity, which increase the risk of allergic sensitisation to aeroallergens. This work has also demonstrated that mechanical obstruction is unlikely to be an important aetiological mechanism in overweight, pre-pubertal children.

## Figures and Tables

**Table 1:** Descriptive comparisons between cases and controls in male and female children: figures shown for cases and controls are mean (s.d.) or number (%)

*Variables*	*Males*			*Females*		
	*Cases (*n*=388)*	*Controls (*n*=388)*	*Differences for the mean or for rates between cases and controls (95% CI)*	*Cases (*n*=244)*	*Controls (*n*=244)*	*Differences for the mean or for rates between cases and controls (95% CI)*
*Measures of obesity*						
Primary measures mean (s.d.)						
BMI (kg/m^2^)	17.0 (3.8)	15.8 (2.5)	1.2 (+0.7, +1.7)^a^	19.0 (4.4)	15.9 (2.1)	3.1 (+2.5, +3.7)^a^
Secondary measures mean (s.d.)						
TSFT (mm)	10.5 (4.2)	9.2 (3.3)	1.3 (+0.8, +1.8)^a^	13.8 (5.3)	10.5 (3.2)	3.3 (+2.6,+4.1)^a^
WC (cm)	56.6 (8.8)	53.6 (6.4)	3 (+1.9, +4.0)^a^	61.0 (10.1)	53.9 (5.8)	7.1 (+5.6, +8.4)^a^
Weight (kg)	26.1 (8.0)	23.9 (5.7)	2.2 (+1.2, +3.2)^a^	30.4 (9.6)	24.1 (5.4)	6.3 (+4.92, +7.69)^a^
						
*Explanatory variables*						
Lung function measures						
FVC (Lt) mean (s.d.)	1.35 (0.36)	1.46 (0.37)	−0.11 (−0.16, −0.06)^a^	1.17 (0.31)	1.22 (0.30)	−0.05 (−0.10, +0.00)
FEV_1_ (Lt) mean (s.d.)	1.15 (0.30)	1.31 (0.34)	−0.16 (−0.21, −0.12)^a^	0.96 (0.27)	1.08 (0.28)	−0.12 (−0.17, −0.08)^a^
PEFR (Lt) mean (s.d.)	2.40 (0.64)	2.72 (0.56)	−0.32 (−0.41, −0.24)^a^	1.77 (0.76)	2.36 (0.57)	−0.59 (−0.70, −0.47)^a^
Sensitisation to one or more allergens (⩾3 mm), *n* (%)						
Cat	216 (56)	55 (14)	42 (+36.0, +48.0)^a^	166 (68)	35 (14)	54 (+46.7, +61.3)^a^
*Dermatophagoides farinae*	99 (25)	21 (5)	20 (+15.2, +24.8)^a^	79 (32)	6 (2)	30 (+23.9, +36.1)^a^
*Dermatophagoides pteronyssinus*	99 (25)	11 (3)	22 (+17.4, +26.6)^a^	91 (37)	16 (6)	31 (+24.3, +37.8)^a^
Grass mix	66 (17)	14 (4)	13 (+8.8, +17.2)^a^	51 (21)	5 (2)	19 (+13.6, +24.4)^a^
Rye	55 (14)	15 (4)	10 (+6.0, +14.0)^a^	83 (34)	2 (1)	33 (+26.9, +39.1)^a^
Tree mix (early blossoming)	70 (18)	13 (3)	15 (+10.8, +19.2)^a^	89 (36)	4 (2)	32 (+27.7, +40.3)^a^
Tree mix (mid-blossoming)	80 (21)	18 (5)	16 (+11.4, +20.6)^a^	81 (33)	3 (1)	30 (+26.0, +38.0)^a^
Weed mix	62 (16)	10 (3)	13 (+9.0, +17.0)^a^	66 (27)	7 (2)	20 (+19.2, +30.8)^a^
Sensitisation to 1⩾allergen	325 (84)	107 (27)	57 (+51.3, +62.7)^a^	234 (96)	54 (22)	74 (+68.2, +79.8)^a^
						
*Potential confounders*						
Father’s age (years) mean (s.d.)	43.3 (8.4)	42.1 (7.8)	1.12 (−0.04, +2.28)	44.1 (9.0)	44.8 (8.9)	0.1 (−5.0, +5.2)
Mother’s age (years) mean (s.d.)	35 (6.1)	34.6 (6.2)	0.36 (−0.51, +1.23)	36.2 (6.0)	36.1 (6.1)	−0.1 (−4.3, +4.1)
Birth weight (kg) mean (s.d.)	2.90 (0.66)	3.00 (0.58)	−0.10 (−0.20, −0.01)^a^	2.94 (0.67)	2.83 (0.62)	0.11 (−0.02, +0.24)
Father’s education (highest qualification), *n* (%)						
None	14 (3.7)	21 (5.6)	−1.9 (−4.9, +1.1)	22 (9.4)	14 (5.9)	3.5 (−1.2, +8.2)
General education	192 (50.8)	198 (53.2)	−2.4 (−1.0, +4.0)	132 (56.2)	156 (65.3)	−9.1 (−8.9, +8.7)
Higher education	172 (45.5)	153 (41.1)	4.4 (−3.0, +11.0)	81 (34.5)	69 (28.9)	5.6 (−2.6, +13.6)
Mother’s education (highest qualification), *n* (%)						
None	14 (3.7)	32 (8.3)	−4.6 (−7.9, −1.3)^a^	26 (10.9)	29 (12.0)	−1.1 (−6.7, +4.5)
General education	208 (54.5)	205 (53.4)	1.1 (−5.9, +8.1)	141 (59.0)	144 (59.8)	−0.8 (−9.5, +7.9)
Higher education	160 (41.9)	147 (38.3)	3.6 (−3.3, +10.5)	72 (30.1)	68 (28.2)	1.9 (−6.2, +10)
Father smoker, *n* (%)						
Yes	129 (33.3)	96 (24.8)	8.5 (+2.1, +14.9)^a^	56 (23.1)	77 (31.8)	−8.7 (−16.6, −0.8)^a^
No	258 (66.7)	291 (75.2)	−8.5 (−14.9, −2.1)^a^	186 (76.9)	168 (68.2)	8.7 (+0.8, +16.6)^a^
Mother smoker, *n* (%)						
Yes	5 (1.3)	1 (0.3)	1.0 (−0.25, +2.25)	4 (1.7)	2 (0.8)	0.9 (−1.1, +2.9)
No	382 (98.7)	385 (99.7)	−1.0 (−2.3, +0.3)	236 (98.3)	241 (99.2)	−0.9 (−2.9, +1.1)
Birth order, *n* (%)						
First child	206 (53.4)	206 (53.1)	0.3 (−6.7, +7.3)	43 (17.8)	47 (19.5)	−1.7 (−8.6, +5.2)
Second child	125 (32.4)	123 (31.7)	0.7 (−5.9, +7.3)	189 (78.1)	180 (74.7)	3.4 (−4.1, +10.9)
Third or greater	55 (14.2)	56 (15.2)	−1.0 (−6.0, +4.0)	10 (4.1)	14 (5.8)	−1.7 (−5.5, +2.1)
Was the child born in Madinah?, *n* (%)						
Yes	332 (85.8)	328 (85.4)	0.4 (−4.5, +5.3)	202 (84.5)	200 (82.6)	1.9 (−4.7, +8.5)
No	55 (14.2)	56 (14.6)	−0.4 (−5.3, +4.5)	37 (15.5)	42 (17.4)	−1.9 (−8.5, +4.7)
Did the mother breastfeed?, *n* (%)						
Yes	290 (75.9)	330 (85.5)	−9.6 (−15.1, −4.1)^a^	208 (86.7)	193 (80.1)	6.6 (0.0, +13.0)
No	92 (24.1)	56 (14.5)	8.5 (+2.1, +14.9)^a^	32 (13.3)	48 (19.9)	−8.7 (−16.6, −0.8)^a^
Medication given in the 1st year of child’s life, *n* (%)						
Paracetamol	367 (95.6)	329 (88.4)	7.2 (+3.4, +11.0)^a^	213 (89.9)	209 (87.4)	2.5 (−3.1, +8.1)
Antibiotics	311 (82.3)	213 (57.9)	24.4 (+18.2, +30.6)^a^	164 (69.5)	148 (61.7)	7.8 (−0.6, +16.2)
Paracetamol administered in the last 12 months, *n* (%)						
Once a week at least	87 (22.9)	70 (18.9)	4.0 (−1.7, +9.7)	54 (22.9)	39 (16.9)	6.0 (−1.1, +13.1)
Once a month at least	220 (57.9)	195 (52.7)	5.2 (−1.8, +12.2)	135 (57.2)	125 (54.1)	3.1 (−5.7, +11.9)
Once a year at least	73 (19.2)	105 (28.4)	−9.2 (−15.2, −3.2)^a^	47 (19.9)	67 (29.0)	−9.1 (−16.7, −1.5)^a^
Exposure to animals, *n* (%)						
Farm animal when mother was pregnant	11 (2.9)	11 (2.9)	0.0 (−2.4, +2.4)	14 (6.1)	8 (3.4)	2.7 (−1.1, +6.5)
Cat at home in the 1st year of child’s life	15 (3.9)	15 (3.9)	0.0 (−2.7, +2.7)	19 (7.9)	6 (2.5)	5.4 (+1.5, +9.3)^a^
Cat at home in the last 12 months	29 (7.5)	25 (6.5)	1.0 (−2.6, +4.6)	18 (7.5)	12 (5.0)	2.5 (+1.2, +8.8)^a^
Farm animal in the 1st year of child life	24 (6.2)	16 (4.2)	2.0 (−1.1, +5.1)	16 (6.8)	5 (2.1)	4.7 (+1.1, +8.3)^a^
Taking exercise per week, *n* (%)						
Never	241 (64.3)	254 (68.8)	−4.5 (−11.1, +2.1)	201 (85.5)	200 (86.2)	−0.7 (−6.9, +5.5)
Once or twice a week	101 (26.9)	86 (23.3)	3.6 (−2.5, +9.7)	31 (13.2)	25 (10.8)	2.4 (−3.4, +8.2)
Three or more a week	33 (8.8)	29 (7.9)	0.9 (−3.0, +4.8)	3 (1.3)	7 (3.0)	−1.7 (−4.3, +0.9)
Watching TV, *n* (%)						
<3 h per day	228 (60.0)	241 (64.6)	−4.6 (−11.4, +2.2)	137 (56.8)	128 (54.9)	1.9 (−6.9, +10.7)
>3 h per day	152 (40.0)	132 (35.4)	4.6 (−2.2, +11.4)	104 (43.2)	105 (45.1)	−1.9 (−10.7, +6.9)
What is the fuel normally used in cooking in your household?, *n* (%)						
Electricity only	20 (5.2)	20 (5.2)	0 (−3.1, +3.1)	9 (3.8)	15 (6.3)	−2.5 (−6.4, +1.4)
Gas only	351 (90.9)	349 (90.4)	0.5 (−3.6, +4.6)	223 (92.9)	220 (92.4)	0.5 (−4.1, +5.1)
Both electricity & gas	15 (3.9)	17 (4.4)	−0.5 (−3.3, +2.3)	8 (3.3)	3 (1.3)	2.0 (−0.7, +4.7)
Air conditioning type, *n* (%)						
Electric fan only	3 (0.8)	1 (0.3)	0.5 (−0.54, +1.54)	1 (0.4)	2 (0.8)	−0.4 (−1.77, +0.97)
Water system only	4 (1.0)	5 (1.3)	−0.3 (−1.8, +1.2)	4 (1.7)	10 (4.1)	−2.4 (−5.4, +0.6)
Freon system only	365 (94.3)	372 (96.6)	−2.3 (−5.2, +0.6)	194 (82.2)	223 (92.5)	−10.3 (−16.1, −4.5)^a^
Both Freon & water systems	15 (3.9)	7 (1.8)	2.1 (−0.2, +4.4)	37 (15.7)	6 (2.5)	13.2 (+8.2, +18.2)^a^
How often does a truck pass through the street adjacent to your home?, *n* (%)						
Rarely	237 (61.6)	268 (70.9)	−9.3 (−15.9, −2.7)^a^	168 (70.3)	169 (70.7)	−0.4 (−8.5, +7.7)
Frequently	148 (38.4)	110 (29.1)	9.3 (+2.7, +15.9)^a^	71 (29.7)	70 (29.3)	0.4 (−7.7, +8.5)
Diet—How many times a week does your child eat the following?, *n* (%)						
Meats						
Never	20 (5.2)	16 (4.2)	1.0 (−4.0, +2.0)	9 (3.7)	26 (10.9)	−7.2 (−11.8, −2.6)^a^
Once or twice a week	110 (28.6)	122 (31.8)	−3.2 (−9.7, +3.3)	83 (34.2)	81 (33.9)	0.3 (−8.1, +8.7)
Three or more a week	254 (66.1)	246 (64.1)	2.0 (−4.7, +8.7)	151 (62.1)	132 (55.2)	6.9 (−1.8, +15.6)
Fruits						
Never	68 (17.7)	44 (11.5)	6.2 (−6.9, +19.3)	29 (12.0)	24 (10.0)	2.0 (−3.5, +7.5)
Once or twice a week	167 (43.4)	175 (45.9)	−2.5 (−9.5, +4.5)	114 (47.1)	115 (48.1)	−1.0 (−9.9, +7.9)
Three or more a week	150 (39.0)	162 (42.5)	−3.5 (−10.4, +3.4)	99 (40.9)	100 (41.8)	−0.9 (−9.6, +7.8)
Rice						
Never	9 (2.4)	8 (2.1)	0.3 (−1.8, +2.4)	6 (2.5)	10 (4.2)	−1.7 (−4.9, +1.5)
Once or twice a week	70 (18.4)	83 (21.7)	−3.3 (−8.9, +2.3)	45 (19.0)	47 (19.7)	−0.7 (−7.7, +6.3)
Three or more a week	301 (79.2)	291 (76.2)	3.0 (−2.9, +8.9)	186 (78.2)	182 (76.2)	2.0 (−5.4, +9.4)
Nuts						
Never	224 (59.4)	227 (60.7)	−1.3 (−8.2, +5.6)	151 (62.9)	116 (50.0)	12.9 (+4.2, +21.6)^a^
Once or twice a week	134 (35.5)	118 (31.6)	3.9 (−2.7, +10.5)	73 (30.4)	93 (40.1)	−9.7 (−18.1,−1.3)^a^
Three or more a week	19 (5.0)	29 (7.8)	−2.8 (−6.2, +0.6)	16 (6.7)	23 (9.9)	−3.2 (−8.1, +1.7)
Fast food						
Never	217 (56.7)	225 (59.2)	−2.5 (−9.4, +4.4)	160 (67.5)	164 (69.8)	−2.3 (−10.5, +5.9)
Once or twice a week	132 (34.5)	122 (32.1)	2.4 (−4.2, +9.0)	60 (25.3)	56 (23.8)	1.5 (−6.1, +9.1)
Three or more a week	34 (8.9)	33 (8.7)	0.2 (−3.8, +4.2)	17 (7.2)	15 (6.4)	0.8 (−3.7, +5.3)

Note: Missing data for individual variables are not shown.

Abbreviations: BMI, body mass index; CI, confidence interval; TSFT, triceps skin fold thickness; WC, waist circumference.

a Statistically significant difference between cases and controls.

**Table 2:** Unadjusted effect of obesity and adiposity measures (TSFT, WC and weight) on the odds of asthma in males and females

*Measures of obesity and adiposity*	*Multiplicative increase in odds of having asthma (95% CI) per unit increase of the independent variable*	
	*Males*	*Females*
*Obesity measure*		
BMI (kg/m^2^)	1.14 (1.08, 1.20)	1.37 (1.26, 1.50)
		
*Adiposity measures*		
TSFT (mm)	1.10 (1.06, 1.15)	1.24 (1.16, 1.33)
WC (cm)	1.06 (1.04, 1.08)	1.14 (1.10, 1.18)
Weight (kg)	1.06 (1.03, 1.08)	1.16 (1.12, 1.20)

Abbreviations: BMI, body mass index; CI, confidence interval; TSFT, triceps skin fold thickness; WC, waist circumference.

**Table 3:** Adjusted ORs (95% CI) for the association between BMI on asthma in males and females

*Independent (X) variables in the multiple logistic regression model*^a^	*ORs of developing asthma (95% CI) adjusted for other factors*	
	*Males*	*Females*
BMI	1.11 (1.03, 1.19)	1.38 (1.23, 1.56)
Birth weight	0.71 (0.49, 1.02)	b
Mother education (none compared with higher)	0.48 (0.16, 1.44)	b
Mother education (general compared with higher)	0.82 (0.51, 1.32)	b
Father smoking	1.17 (0.76, 1.80)	0.45 (0.22, 0.93)
Breastfed	0.74 (0.44, 1.26)	3.62 (1.42, 9.25)
Paracetamol given in the 1st year of child’s life	1.66 (0.67, 4.09)	b
Antibiotics given in the 1st year of child’s life	2.45 (1.54, 3.91)	b
Paracetamol given to the child in the last 12 months (once a week compared with yearly)	1.94 (1.00, 3.80)	7.09 (2.48, 20.8)
Paracetamol given to the child in the last 12 months (once a month compared with yearly)	1.90 (1.14, 3.14)	2.23 (1.05, 4.72)
Cat at home in the 1st year of child’s life	c	0.83 (0.10, 7.28)
Cat at home in the last 12 months	c	2.25 (0.42, 12.1)
Farm animal in the 1st year of child’s life	c	2.56 (0.36, 18.4)
Air conditioning type used at home (electric fan compared with Freon system)	c	3.58 (0.02, 529)
Air conditioning type used at home (water system compared with Freon system)	c	0.51 (0.09, 2.96)
Air conditioning type used at home (both Freon and water system compared with Freon system)	c	19.4 (3.56, 106)
Frequency of a truck passing through the street adjacent to the home (frequently compared with rarely)	1.39 (0.89, 2.78)	b
Eating meat (once or twice a week compared with never)	c	10.2 (2.03, 51.2)
Eating meat (three times a week or more compared with never)	c	8.68 (1.83, 41.0)
Eating nuts (once or twice a week compared with never)	c	0.55 (0.29, 1.04)
Eating nuts (three times a week or more compared with never)	c	0.80 (0.27, 2.37)

Abbreviations: BMI, body mass index; CI, confidence interval; OR, odds ratio.

a Only those variables that remained in the model in the last step of the backward stepwise regression process are displayed in the table.

b Not in the model for females.

c Not in the model for males.

**Table 4:** Estimates (95% CIs) for the coefficient of BMI in conditional logistic regression analysis adjusted for selected lung function and sensitisation and also for other factors found to be associated with obesity in Table 1

*Lung and sensitisation factors*	*Males*	*Females*
None	1.11 (1.03, 1.19)	1.38 (1.23, 1.56)
FEV_1_	1.10 (1.02, 1.18)	1.37 (1.22, 1.54)
FVC	1.11 (1.03, 1.19)	1.40 (1.24, 1.57)
Sensitisation to more than one allergen	1.09 (0.99, 1.19)	1.25 (0.96, 1.60)
FEV_1_+sensitisation to more than one allergen	1.09 (0.98, 1.19)	1.28 (0.96, 1.69)
FVC+sensitisation to more than one allergen	1.09 (0.99, 1.19)	1.25 (0.97, 1.61)

Abbreviations: BMI, body mass index; CI, confidence interval; FEV_1_, forced expiratory volume in 1 s; FVC, forced vital capacity.

## Notes

AS is joint Editor-in-Chief of npjPCRM, but was not involved in the editorial review of, nor the decision to publish, this article. None of the other authors report any conflict of interest.
